# Heterogeneous Coordination Environments in Lithium-Neutralized Ionomers Identified Using ^1^H and ^7^Li MAS NMR

**DOI:** 10.3390/ma5081508

**Published:** 2012-08-23

**Authors:** Todd M. Alam, Janelle E. Jenkins, Dan S. Bolintineanu, Mark J. Stevens, Amalie L. Frischknecht, C. Francisco Buitrago, Karen I. Winey, Kathleen L. Opper, Kenneth B. Wagener

**Affiliations:** 1 Department of Nanostructured and Electronic Materials, Sandia National Laboratories, Albuquerque, NM 87185, USA; E-Mail: jejenki@sandia.gov; 2 Department of Nanoscale and Reactive Processes, Sandia National Laboratories, Albuquerque, NM 87185, USA; E-Mail: dsbolin@sandia.gov; 3 Computational Materials Science and Engineering Department, Sandia National Laboratories, Albuquerque, NM 87185, USA; E-Mails: msteve@sandia.gov (M.S.); alfrisc@sandia.gov (A.F.); 4 Department of Chemical and Biomolecular Engineering, University of Pennsylvania, Philadelphia, PA 19104, USA; E-Mail: buitrago@seas.upenn.edu; 5 Dept. of Material Science and Engineering, University of Pennsylvania, Philadelphia, PA 19104, USA; E-Mail: winey@seas.upenn.edu; 6 DuPont Central Research and Development, Wilmington, DE 19880-0302, USA; E-Mail: Kathleen.Opper@usa.dupont.com; 7 Center for Macromolecular Science and Engineering, Department of Chemistry, University of Florida, Gainesville, FL 32611, USA; E-Mail: wagener@chem.ufl.edu

**Keywords:** MAS NMR, REDOR, lithium ionomer, precise polymer, ionomer

## Abstract

The carboxylic acid proton and the lithium coordination environments for precise and random Li-neutralized polyethylene acrylic acid P(E-AA) ionomers were explored using high speed solid-state ^1^H and ^7^Li MAS NMR. While the ^7^Li NMR revealed only a single Li coordination environment, the chemical shift temperature variation was dependent on the precise or random nature of the P(E-AA) ionomer. The ^1^H MAS NMR revealed two different carboxylic acid proton environments in these materials. By utilizing ^1^H-^7^Li rotational echo double resonance (REDOR) MAS NMR experiments, it was demonstrated that the proton environments correspond to different average ^1^H-^7^Li distances, with the majority of the protonated carboxylic acids having a close through space contact with the Li. Molecular dynamics simulations suggest that the shortest ^1^H-^7^Li distance corresponds to un-neutralized carboxylic acids directly involved in the coordination environment of Li clusters. These solid-state NMR results show that heterogeneous structural motifs need to be included when developing descriptions of these ionomer materials.

## 1. Introduction

Ionomers are polymers containing low concentrations (<15 mol%) of ionic groups covalently bonded to the backbone. These materials are currently being developed for a wide range of applications including ion transport membranes and solid electrolytes for fuel cells and batteries, biological compatible polymers and adhesives for biomedical applications, and ionomer adhesive resins for flexible packaging films and lamination of safety glasses and solar devices [1,2,3,4]. 

Current research in this laboratory is directed towards understanding the local structure and morphology of ionomers, and how these properties impact the material performance. Even subtle changes, such as changing from random to precise placement of the ionic groups along the polymer backbone, can produce significant changes in the performance. For example, proton exchange membranes (PEM) with precise sulfonic acid group placement, versus a statistical random distribution, show increased conductivity due to changes in the ionic cluster distributions and the formation of water channels allowing for improved proton transport [5]. Precise ionomer materials have been developed through refinement of the acyclic diene metathesis (ADMET) polymerization chemistry to produce polyethylene (PE) materials with different functional groups precisely placed along the backbone, including the incorporation of alkyl chains, halogens, phosphonic acid, carboxylic acid, methylimidazolium, and amine groups [6,7,8,9,10,11]. Nucleophilic polycondensation of biphenol monomers with sulfonated dihalides allows the controlled production of precise singlet or doublet sulfonic acids [5]. 

**Chart 1:**
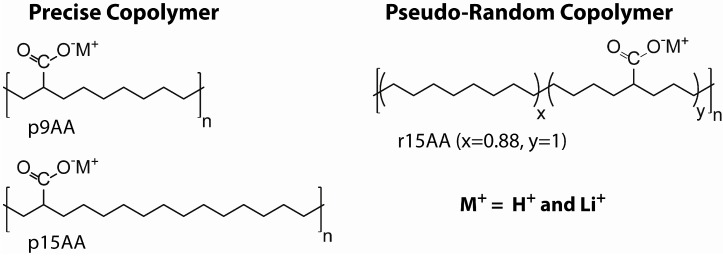
Structures of the precise and pseudo-random Li-neutralized poly(ethylene-*co*-acrylic) acid copolymers ionomers.

In this paper, the ^1^H and ^7^Li magic angle spinning (MAS) nuclear magnetic resonance (NMR) is presented for partially Li-neutralized poly(ethylene-*co*-acrylic acid) P(E-AA) ionomers. The precise PE ionomers have been extensively characterized using X-ray scattering, scanning electron microscopy, NMR [7,12]. The crystallization and segmental dynamics as a function of random versus precise functional group spacing has been probed using ^13^C, ^19^F and ^31^P MAS NMR [7,8,13,14], high-speed ^1^H MAS NMR [15], and static ^2^H NMR [9]. For the present study, characterization of the heterogeneous carboxylic acid proton environments observed for both the precise and random P(E-AA) materials is of interest. The chemical structures of the target materials are shown in Chart 1, and include the precise (p) copolymers with the acid group spaced exactly 9 carbon atoms apart and 43% Li neutralization (p9AA-43%Li), the precise copolymer with 15 carbon atom spacing and 45% Li neutralization (p15AA-45%Li), and a pseudo-random (r) material where the average carbon spacing is 15 atoms and 31% Li neutralization (r15AA-31%Li). Additional details about the preparation of these ionomers are provided in the experimental section. 

## 2. Results and Discussion

### 2.1. Solid-State ^1^H MAS NMR

**Figure 1:**
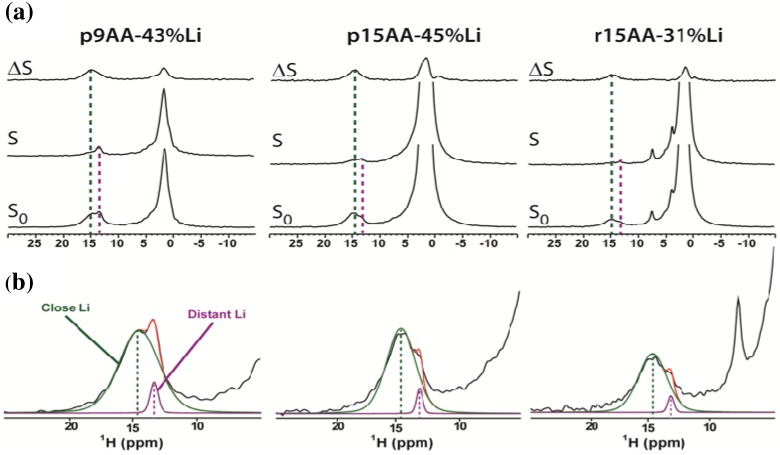
(**a**) The solid-state ^1^H MAS REDOR NMR of the partially Li-neutralized precise and pseudo-random P(E-AA) ionomers for $\tau_{\text{REDOR}}$ = 1.8 ms. The reference spectra are denoted by **S_0_**, the ^1^H-^7^Li-dipolar coupled dephased spectra are labeled **S**, and the differences spectra by **ΔS**; (**b**) The carboxylic acid spectral expansion of the reference spectra showing deconvolutions into two different environments corresponding to a “close” and “distant” ^1^H-^7^Li distance.

The ^1^H MAS NMR spectra for the Li-neutralized P(E-AA) ionomers has previously been discussed [15], but will be briefly summarized (Figure 1, S_0_ spectra). The dominant set of overlapping resonances between *δ* = +1.5 and +2.5 ppm correspond to the methylene (CH_2_) and methine (CH) protons of the polymer backbone, while the smaller resonances between *δ* = +13.4 and +14.7 ppm are assigned to the COOH of the acrylic acid side chain. The primary focus will be the characterization of these acid proton environments. In the un-neutralized P(E-AA) copolymers only a single COOH environment was observed between *δ* = +12.3 and +12.4 ppm (332K), or between *δ* = +12.7 and 13.2 ppm (294K) [15], and is due to formation of a hydrogen bonded cyclic acid dimer. The chemical shift assignment of this dimer is consistent with the +12.9 ppm (324 K) recently reported for poly(methacrylic acid) hydrogen complexes [16], and the +13.1 ppm for acetic acid dimers formed in a Freon mixture (110K) [17]. In polymer systems the ^1^H NMR chemical shift for an isolated, non-hydrogen bonded, COOH group is *δ* = +9 ppm, while water protons in exchange with this isolated acid are at *δ* = +6.5 ppm [16]. Neither the isolated COOH or exchanging water environments were detected in the P(E-AA) materials. The temperature dependence of the ^1^H NMR chemical shift for the COOH dimer in the unneutralized materials reflects changes in the hydrogen bond strength due to polymer dynamics, and has been previously discussed (see Figure 3 of [15]). In the Li-neutralized P(E-AA) ionomers the COOH dimer resonance disappears entirely, and is replaced by two overlapping carboxylic acid proton resonances at *δ* = +13.4 and +14.7 ppm (294 K). The ^1^H NMR chemical shifts, line widths, and relative percentages of these two new acid proton environments are given in Table 1.

**Table 1:** ^1^H and ^7^Li magic angle spinning (MAS) nuclear magnetic resonance (NMR) chemical shifts, line widths, and relative fractions of the acid proton and lithium environments in the random and precise Li-neutralized P(E-AA) ionomers.

Sample	*T*_g_ (K)	*δ*(^1^H) (ppm) ^a^	FWHM (^1^H) (Hz) ^a^	Fraction (%) ^a^	*δ*(^7^Li) (ppm) ^b^	FWHM (^7^Li) (Hz) ^b^
p9AA-43%Li	346	+14.7 +13.4	2050 520	89% 11%	0.20	280
p15AA-45%Li	347	+14.8 +13.3	1680 360	94 6	0.21	290
r15AA-31%Li	313 (amorphous) 344 (crystalline)	+14.9 +13.3	1790 360	94 6	0.35	265

^a^ :^1^H MAS NMR results obtained at 294 K using a 30 kHz spinning speed; ^b^ :The ^7^Li MAS NMR chemical shift reported for 315 K.

#### 2.1.1. Hydrogen Bond Correlations

The increase in the acid ^1^H NMR chemical shifts for the Li-neutralized ionomers reveals a strengthening of the hydrogen bond strength in comparison to the COOH cyclic dimer. Correlations between the ^1^H NMR chemical shift and hydrogen bond length have been put forward by several groups [18,19,20]. These types of correlations also include the work of Limbach and co-workers [17,21,22], which relate the NMR chemical shift to the hydrogen bond order using the coordinates:

(1) $$q_{1}=\frac{1}{2}\left(r_{1}+r_{2}\right),\;q_{2}=r_{1}+r_{2}$$

where *r*_1_ and *r*_2_ are the OH and HO bond distances, respectively. For a linear hydrogen bond *q*_1_ represents the displacement of the hydrogen from the center (asymmetry), and *q*_2_ corresponds to the O•••O distance. For carboxylic acids, the ^1^H NMR chemical shift as a function of the *q*_1_ coordinate is given by [21]

(2) $$\delta_{1_{H}}(\text{ppm})=6+14.5\;\text{exp}(-6.2q_{1}^{2})$$

**Figure 2:**
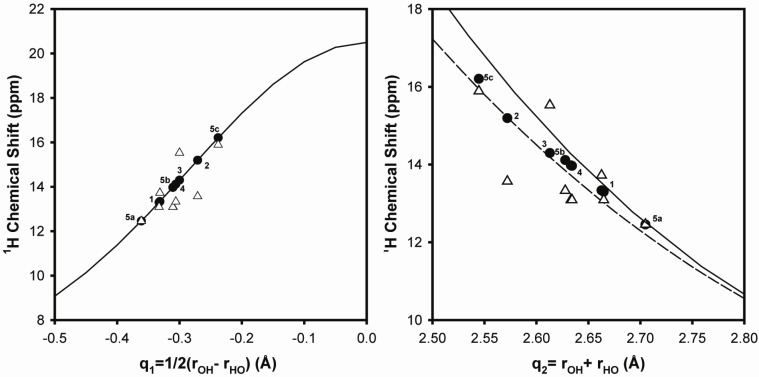
^1^H NMR chemical shift correlations for both the *q*_1_ and *q*_2_ hydrogen bond coordinates. Chemical shifts obtained using either Equation 2 (●) or with Gaussian *ab initio* methods (Δ) for the different model hydrogen bonded complexes 1–5 (shown in Figure 3). For the *q*_2_ correlation both the parameter sets of Tolstoy *et al.* [17] (solid line) and Steiner [23] (dashed line) are presented.

Relationships between *q*_1_ and *q*_2_ have also been previously described,

(3) $$q_{2}=2r^{o}-2q_{1}+2b\;\text{ln}\left(1+\text{exp}\left(2q_{1}/b\right)\right)$$

where the parameter *r*^o^ is the OH distance in a free acid, and *b* describes the decay of bond order with increasing distance. The selection of these parameters varies with the types of hydrogen bonds and the temperature range investigated. Tolstoy *et al.* [17] reported the parameter set *b* = 0.467 Å and *r*^o^ = 0.902 Å, while Steiner [23] obtained *b* = 0.397 Å and *r*^o^ = 0.925 Å for OHO hydrogen bonds at room temperature. Using Equations (1–3), correlations between the ^1^H NMR chemical shift and *q*_1_ and *q*_2_ were obtained, and are shown in Figure 2 for both of the *b* and *r*^o^ parameter sets. Regardless of the parameter set used, an increase in the ^1^H chemical shift reflects: (1) a decrease in *q*_1_, representing a reduction in the hydrogen bond asymmetry and an increase in the hydrogen bond strength, and (2) a decrease in *q*_2_, representing a shortening of the hydrogen bond length. Therefore, in the Li-neutralized ionomers, there is an increase in the hydrogen bond strength with neutralization in comparison to the un-neutralized cyclic dimers. The change in the chemical shift (Δ*δ* ~ +2.4 ppm) represents a reduction of the O•••O bond length by ~0.1 Å. 

**Figure 3:**
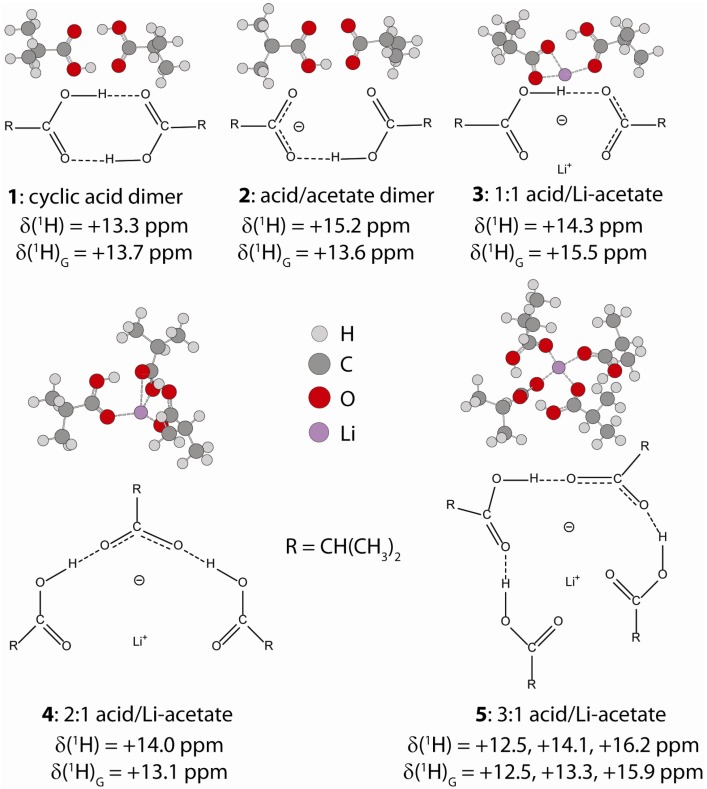
Structures of the model isobutyric/Li acetate hydrogen bond complexes, and the ^1^H NMR chemical shift predicted using either Equation 2 (*δ*) or with Gaussian *ab initio* methods (*δ*_G_).

The variation of the ^1^H NMR chemical shift and the increase in the hydrogen bond strength can also be discussed in terms of the formation of hydrogen bond complexes (or clusters) with Li neutralization. Tolstoy and co-workers have used NMR to characterize the hydrogen bond strength in acetic acid and acetate complexes formed with the addition of tributylammonium (TBA+) acetate. These structures included the acetic acid cyclic dimer, 1:1, 1:2 and 1:3 acetate/acid complexes. They observed a large variation in the ^1^H chemical shift and demonstrated that the variation can be described by the correlations given in Equations 2 and 3. 

For the P(E-AA) ionomers distinct hydrogen bonded complexes could not be isolated, so instead we have elected to predict the ^1^H NMR chemical shifts for a series of different model Li^+^-isobutyric acid complexes (Figure 3). Using structures optimized with a PCM solvent (assuming a dielectric of *ε* = 2.3 to represent the surrounding continuum of the PE polymer), the ^1^H NMR chemical shifts were estimated using Equations 2 and 3, and are shown as solid symbols (●) in Figure 2. The ^1^H chemical shifts (*δ*_G_) can also be predicted with *ab initio* methods using the Gaussian software package, and are given by the open triangles (Δ) in Figure 2. For the complexes **1** through **4**, one distinct proton environment was predicted, while for the 1:3 acid/Li acetate complex (**5**) three different ^1^H chemical shifts were obtained. These predicted chemical shifts for the model clusters span a relatively small range on the *q*_1_ and *q*_2_ correlations. It was also noted that the *q*_2_ correlation utilizing the Steiner [23] parameter (*b* = 0.397 Å and *r*^o^ = 0.925 Å) had an improved fit to the predicted ^1^H chemical shifts for these model clusters. The Gaussian predicted chemical shifts showed similar trends, but revealed a larger degree of scatter for both the *q*_1_ and *q*_2_ correlations, and most likely reflects the small size of the model clusters used in the predictions.

In the acetic acid studies [17], the formation of the 1:1 acid-acetate hydrogen bonded complex lead to a large initial increase in the ^1^H chemical shift (Δ*δ* ~ +6.1 ppm) corresponding to stronger hydrogen bonding. With increasing complex size there was a subsequent decrease in the observed chemical shift. In contrast, for the model isobutyric acid complexes studied here a much smaller predicted chemical shift variation (Δ*δ* ~ +1 ppm) between the cyclic dimer and the 1:1 acid/acetate complex (**3**) was observed. In addition, the model 3:1 complex (**5**) had distinct proton environments with the chemical shift ranging between +15.9 and +12.5 ppm (Figure 3), giving an average of +14.3 ppm. Unfortunately the narrow chemical shift variation predicted for formation of the different isobutyric acid/Li-acetate hydrogen bond complexes does not allow specific details about the size or relative population of the hydrogen bond structures within the P(E-AA) ionomers to be elucidated. 

### 2.2. ^1^H-^7^Li REDOR MAS NMR 

To further explore the local coordination environment of these different carboxylic acid protons a series of ^1^H-^7^Li rotational echo double resonance (REDOR) MAS NMR spectra were obtained, and are shown in Figure 1a. The difference spectra is defined as $∆S=\;S_{0}-\;S$ where S is the spectra under the influence of the REDOR pulse sequence that recouples the heteronuclear ^1^H-^7^Li dipolar coupling, and $S_{0}$ is the reference spectra without the REDOR recoupling sequence. The heteronuclear dipolar Hamiltonian oscillates sinusoidally under MAS and is effectively averaged away during a rotor period. The introduction of a π pulse to the ^7^Li nuclei during the REDOR pulse sequence inverts the sign of the heteronuclear dipolar coupling and recouples this interaction. The spectral intensity of proton environments that have significant ^1^H-^7^Li dipolar coupling are reduced due to dipolar dephasing and give rise to significant buildups in the ΔS difference spectra. Additional experimental details are provided in the experimental section. Inspection of the REDOR spectra reveal that the different carboxylic acid proton environments can be separated based on differential ^1^H-^7^Li dipolar dephasing responses. Recall that for a fully neutralized acid group there is no proton, and it would not be observed in the ^1^H MAS NMR spectra. The high chemical shift acid environment (*δ* = +14.7) has a larger ^1^H-^7^Li dipolar coupling as seen from the significant ΔS dephasing difference. The minor carboxylic acid environment (*δ* = +13.4 ppm) has a reduced ΔS dephasing spectra intensity, corresponding to a smaller ^1^H-^7^Li dipolar coupling. These different environments will be denoted as “close” and “distant” with respect to Li coordination for the remainder of the paper (Figure 1). A ^1^H-^7^Li dipolar contact between the Li and the protons in the methylene or methine of the polymer backbone was also observed. Because the numerous backbone CH_2_ and CH resonances overlap and are unresolved in the ^1^H MAS NMR spectra, these ^1^H-^7^Li interactions are not discussed further. 

#### 2.2.1. Measuring the Average ^1^H-^7^Li Distance

**Figure 4:**
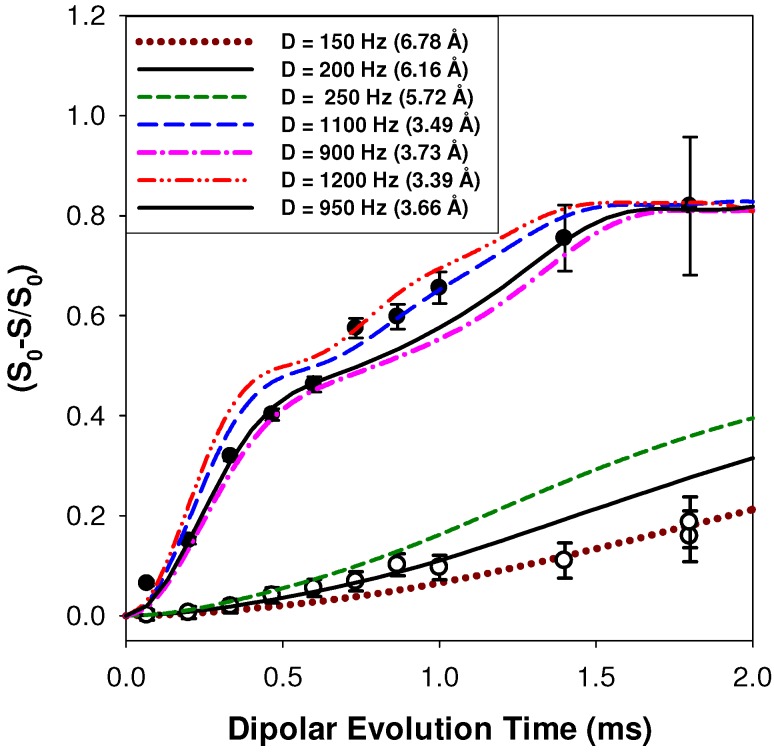
^1^H-^7^Li REDOR MAS NMR buildup curves for the carboxylic acid proton environments at *δ* = +14.7 ppm (●) and *δ* = +13.4 ppm (○) in the p9AA-43%Li ionomer material.

The ^1^H-^7^Li REDOR MAS NMR response for the p9AA-43%Li ionomer with increasing recoupling times is shown in Figure 4, and was used to extract the dipolar coupling strength based on a 2-spin simulation. The inter-nuclear distance was then calculated from the measured ^1^H-^7^Li dipolar coupling (*D*) using:

(4) $$D_{ij}=\frac{\mu_{0}\hbar \gamma_{i}\gamma_{j}}{4\pi r_{ij}^{3}}$$

where $r_{ij}$ is the distance between the ^1^H and the ^7^Li nucleus; $\gamma_{i}$ and $\gamma_{j}$ are the gyromagnetic ratios of the coupled nuclei; $\mu_{0}$ is the vacuum permeability; and ℏ is the Plank constant. For the p9AA-43%Li ionomer, the average ^1^H-^7^Li distance of the deshielded *δ* = +14.7 ppm proton environment was determined to be 3.65 ± 0.2 Å, while the *δ* = +13.4 ppm proton environment revealed an average ^1^H-^7^Li distance of 6.2 ± 0.5 Å. The initial 0.5 ms section of REDOR buildup curve was used for distance evaluation, since this region of the dephasing response is known to be dominated by the magnitude of the dipolar coupling (discussed in experimental section). Multiple simulation curves for other distances are shown to provide a measure of the accuracy of the extracted distance. All simulations were corrected for the natural abundance of ^7^Li. The fraction weighted average REDOR distance for the P9AA-43%Li ionomer material was ~3.9 Å.

**Figure 5:**
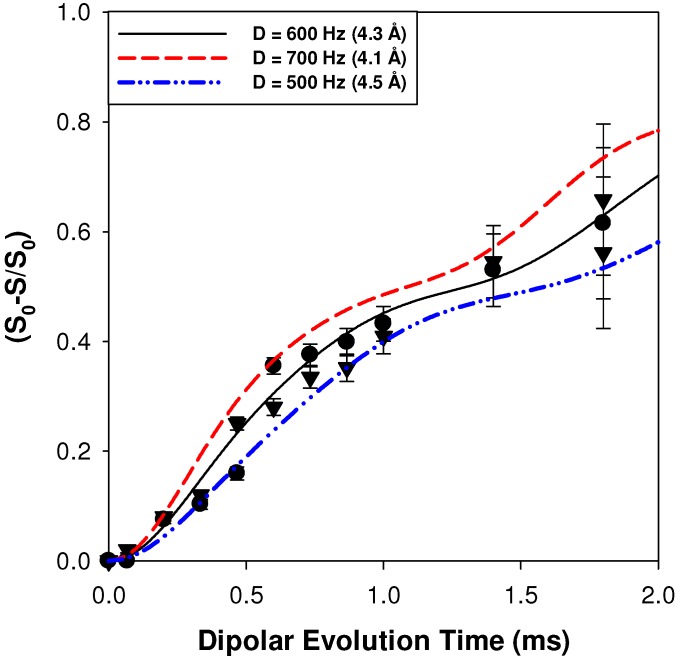
^1^H-^7^Li REDOR MAS NMR buildup curves for the average carboxylic acid proton environment in the p15AA-45%Li (●) and r15AA-31% (▼) ionomer material.

The ^1^H-^7^Li REDOR MAS NMR buildup curves for the p15AA-45%Li and the r15AA-31%Li ionomer materials are shown in Figure 5. For these materials the individual proton environments were not as cleanly resolved during the REDOR experiments due to the reduced intensity of the more shielded component. Therefore, the REDOR buildup curves were obtained by integration of the entire carboxylic acid region, and represent an average of all ^1^H-^7^Li acid distances present in the ionomer materials. For both the p15AA and r15AA ionomers an average distance of 4.3 ± 0.2 Å was determined, which is longer than the weighted average distance measured for the p9AA-43%Li ionomer. The REDOR NMR results show that the local distribution of the Li around the protons of the carboxylic acids are very similar for the precise and random 15-carbon materials, and that increasing the spacer length produces an increase in the average ^1^H-^7^Li distance in comparison to the p9AA ionomer. 

#### 2.2.2. MD Structural Motifs

**Figure 6:**
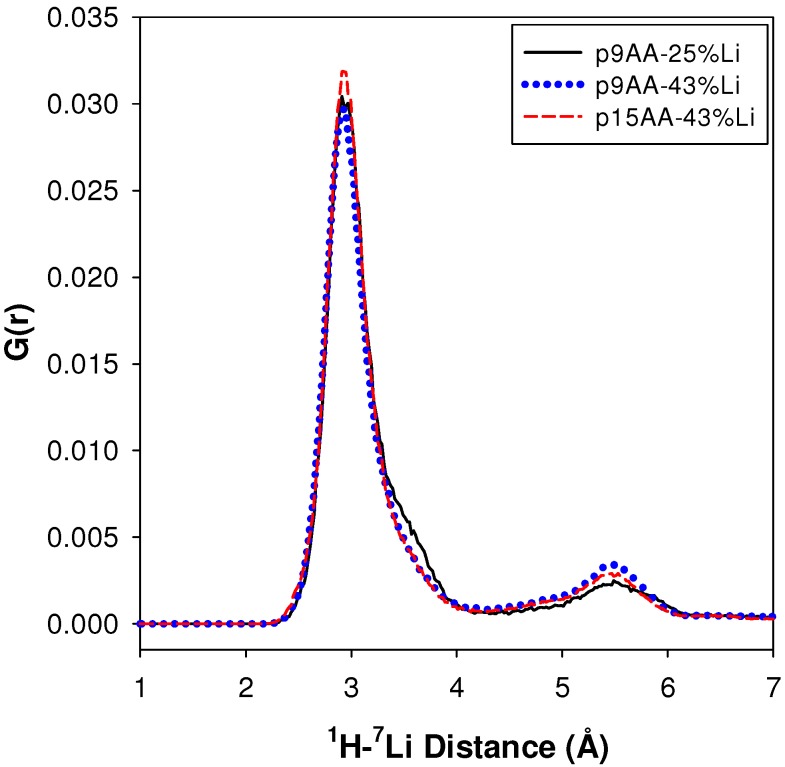
^1^H-^7^Li pair correlation functions for different P(E-AA) ionomers predicted from molecular dynamics (MD) simulations (423 K).

A description of the structural motif that gives rise to the dominant (>89%) carboxylic acid environment with the relatively short ^1^H-^7^Li distance is still needed. To explore possible structures, a series of molecular dynamics (MD) simulations of the P(E-AA) ionomers were analyzed. Figure 6 shows the ^1^H-^7^Li pair distribution function G(r) predicted from the MD simulations for different precise spacing lengths and Li neutralization levels. Two distinct maximums are observed, with the major peak having a ^1^H-^7^Li distance of ~2.9 Å, and the smaller component with a ^1^H-^7^Li distance of approximately 5.4 Å. Table 2 summarizes these G(r) results. The peak maxima and relative fractions are predicted not to change significantly between the p9AA and p15AA material, or with the degree of Li neutralization changing from 25 to 43%. The observation of two distinct ^1^H-^7^Li environments corresponds well with the two different distances determined from the experimental REDOR NMR results (Figure 4). The experimental ^1^H-^7^Li distances for both acid environments are about 0.5 Å longer than predicted in the MD simulation. The longer experimental distances may be a result from the elevated temperature used for the MD simulations (423 K), or may result from partial averaging of the ^1^H-^7^Li dipolar coupling by local chain motions not quenched at the experimental temperature (294 K). The MD simulations also predict that the environment with the shortest ^1^H-^7^Li distance is the dominant fraction (84%), which agrees very well with the 89% determined experimentally. The weighted average ^1^H-^7^Li distances (~3.4 Å) obtained from the MD simulation (Table 2) do not compare as well to the experimental 4.3 Å obtained for the unresolved REDOR measurements (Figure 5). While the MD simulations are for the Li-neutralized ionomers in a melt, they do provide a qualitative structural picture of the different acid environments that may exist in these partially neutralized ionomers. 

**Table 2:** Maximum, relative fraction and average distance for the different ^1^H-^7^Li environments predicted from MD simulations.

Sample	^1^H-^7^Li (Å)	Major Fraction(%) ^a^	^1^H-^7^Li (Å)	Minor Fraction (%) ^a^	<^1^H-^7^Li> (Å)
p9AA-25% Li	2.91	84	5.49	16	3.43
P9AA-43% Li	2.93	80	5.43	20	3.49
p15AA-43% Li	2.95	83	5.53	17	3.42

^a^ : G(r) integration regions were between 0 and 4 Å, and 4 and 7 Å.

**Figure 7:**
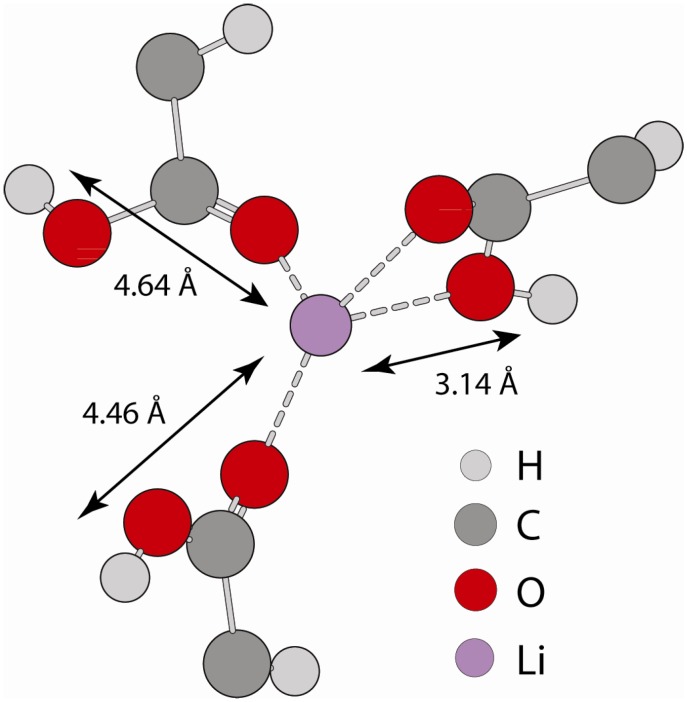
Extracted MD snap shot showing an example of the different ^1^H-^7^Li bonding environments, and the corresponding ^1^H-^7^Li distance.

Figure 7 shows a MD snap shot that provides an example of the different ^1^H environments giving rise to the distinct ^1^H-^7^Li distances observed in G(r). The typical coordination sphere for Li involves four oxygen atoms, but these oxygen atoms can be from either fully non-protonated (fully neutralized) carboxylic acid groups, or un-neutralized (protonated) carboxylic acids, or some combination of the two different oxygen types. The snap shot in Figure 7 shows coordination involving only unneutralized carboxylic acids. The short ^1^H-^7^Li G(r) distance results from carboxylic acid protons that are attached to oxygen atoms directly coordinated to Li, while the longer ^1^H-^7^Li distance results from protons attached to oxygen atoms that are not within the first coordination sphere of Li, but are instead coordinated to other nearby acid groups. Inspection of the MD simulations show that the majority of the acids, whether neutralized or unneutralized, are involved in the formation of extended Li clusters, consistent with the structural picture emerging from X-ray scattering and simulations [24,25]. There is a small fraction of acid groups on the edge of clusters where the corresponding protons are not spatially near Li. In addition, the MD simulations did not reveal the formation of isolated hydrogen bonded acid-acid dimers as observed in the un-neutralized P(E-AA) copolymers, which is consistent with the experimental ^1^H MAS NMR results. The MD simulations also support the experimentally observed disappearance of the *δ* = +12.3 ppm dimer acid resonance with the addition of Li. It should be noted that the distances extracted from the REDOR experiments assumed a 2-spin approximation (^1^H-^7^Li) and that with the formation of extended Li-Li clustering the carboxylic acid protons would be expected to have dipolar interactions with multiple Li nuclei. These multi-spin interactions would give rise to an increase in the dipolar dephasing rate, such that the simulated REDOR ^1^H-^7^Li distance would be shorter than the actual distance. Regardless of the approximation employed, it is clear that the two different ^1^H environments resolved have different dipolar interaction strengths with Li. 

### 2.3. ^7^Li MAS NMR 

**Figure 8:**
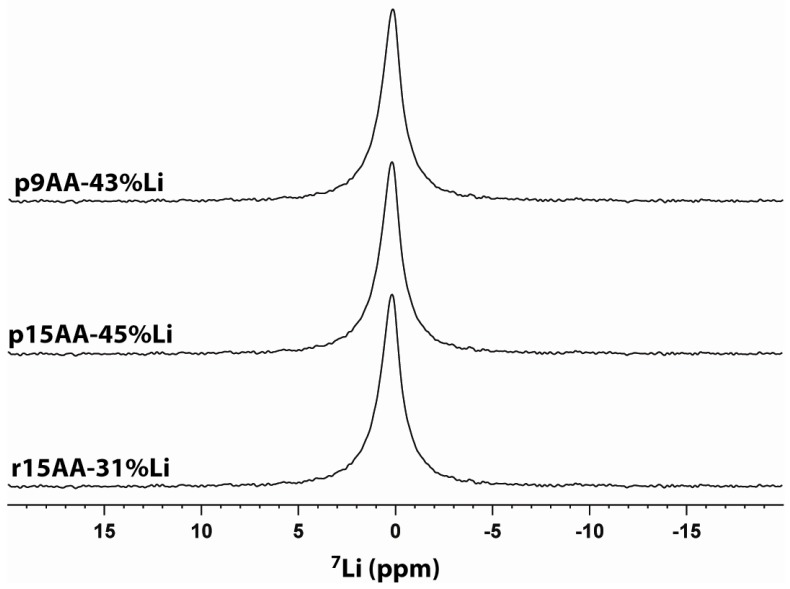
^7^Li MAS NMR of partially Li-neutralized P(E-AA) ionomers.

The ^7^Li MAS NMR spectra for the different ionomer materials are shown in Figure 8. Only a single resonance was observed for each sample. Different Li coordination environments were not resolved. The chemical shifts and line widths are summarized in Table 1. The resolution of ^7^Li MAS NMR is typically poorer than ^6^Li MAS NMR due to residual ^7^Li-^7^Li dipolar couplings and larger ^7^Li quadrupolar coupling constants (QCC), but ^6^Li has the lower natural abundance (7.4%) and a lower observe frequency. Due to the limited sample size available (4–10 mg ionomer), we were unable to obtain ^6^Li MAS NMR spectra for these materials. 

#### 2.3.1. Temperature Variation

**Figure 9:**
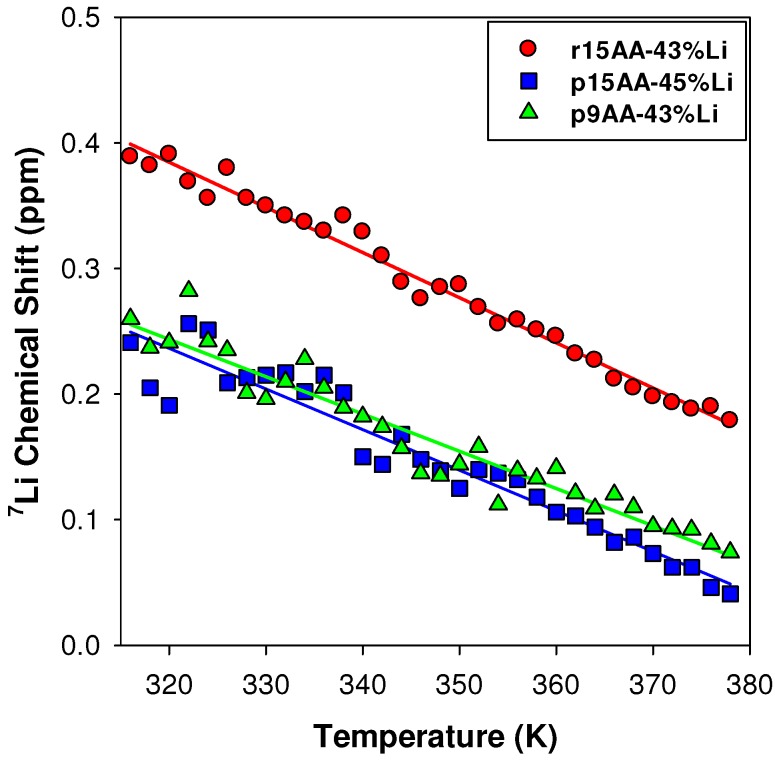
Temperature variation of the ^7^Li MAS NMR chemical shifts for different partially Li-neutralized P(E-AA) ionomers.

The observed ^7^Li NMR chemical shift temperature variations (Figure 9), and relatively narrow line widths suggest some Li mobility within these materials. For all of the ^7^Li ionomer materials studied, increasing the temperature produces a decrease in the chemical shifts, reflecting changes in the local Li coordination environment. The temperature variation for the p9AA-43%Li and p15AA-45%Li ionomers are very similar. The r15AA-31% ionomer has a similar temperature variation, but has a chemical shift offset (Δ*δ* ~ 0.15 ppm). This offset is attributed to differences in the percent crystalline component, with the p9AA and p15AA ionomer materials being entirely amorphous, and the r15AA ionomer being semi-crystalline, with two different endothermic transitions (Table 1). 

#### 2.3.2. *Ab Initio* Prediction of ^7^Li NMR Chemical Shifts

**Figure 10:**
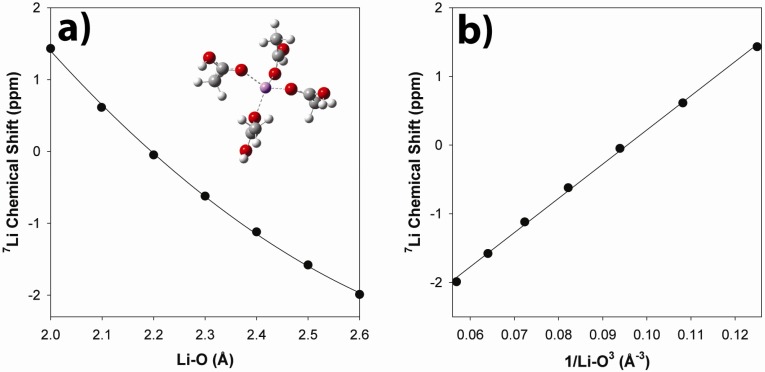
*Ab initio* calculations of the ^7^Li NMR chemical shift variation of the a) Li-O and b) (Li-O)^−3^ distances in the tetra-acetic acid-Li cluster.

*Ab initio* chemical shift calculations were used to evaluate the impact of bond length changes within the Li coordination sphere on the observed ^7^Li NMR chemical shift, and how these could be correlated to the observed chemical shift temperature variation. Calculations were performed on an isolated model cluster involving 4 acetic acids directly coordinated to Li (Inset Figure 10). From the MD simulations presented above, the Li coordination environments were found to be a mixture of Li surrounded entirely by acetate type anions (unprotonated, fully neutralized), a combination of unprotonated and protonated coordination acids, and Li sites involving entirely protonated (unneutralized) coordinating environments. While the model cluster is a very simplistic, it represents a limiting case of the Li coordination environment involving only un-neutralized acid species, and provides a measure on the impact of bond length variations on the chemical shift. The ^7^Li NMR chemical shift as a function of Li-O bond distance is shown in Figure 10a. Previous studies have shown that a linear variation is observed as a function of 1/r(Li-O)^3^ [26], as seen in Figure 10b.

Based on this simple bond length correlations, the observed ^7^Li MAS NMR chemical shift suggest an average Li-O bond distance near 2.15 Å, which is similar to the 2 Å maximum observed in the Li-O G(r) obtained from MD simulations [27]. Experimentally the ^7^Li NMR chemical shift becomes smaller (more shielded) with increasing temperature. This can be envisioned as resulting from a lengthening of the average Li-O coordination distance at higher temperatures. The chemical shift temperature variation is small, changing by only −0.15 ppm over a 60 °C increase. This variation would result from a 0.01 Å increase in the Li-O bond distance.

### 2.4 Summary of Local Structure and Impact on T_g_

These NMR results demonstrate a distinct change in the local carboxylic acid environment with Li neutralization. For the pure unneutralized P(E-AA) copolymers, the carboxylic acids form predominantly cyclic dimer structures, with a relatively low *T*_g_ that varies with acid group spacing. This *T*_g_ variation reflects the energetic competition between hydrogen bonded dimer formation and chain packing in the amorphous phase [15]. With partial Li neutralization, the cyclic dimer environment completely disappears (is not simply fractionally reduced), and is replaced by carboxylic acid hydrogen bonded complexes that are involved in Li coordination. This cation coordination, or ionic cross-linking, results in *T*_g_ values for the amorphous precise ionomers that are 50^o^ to 70^o^ higher versus the unneutralized copolymers. The lack of a crystalline phase in the p9AA and p15AA copolymers has been previously quantified using solid-state ^13^C MAS NMR [14]. The amorphous nature of the p9AA-43%Li and p15AA-45%Li ionomer was similarly confirmed using ^13^C MAS NMR (data not shown). 

The ^7^Li NMR chemical shift results suggest that the local cation coordination is very similar between the p9AA and p15AA ionomers, while the ^1^H-^7^Li REDOR NMR results reveal that the carboxylic acid environments are heterogeneous. The dominant acid environment reveals a shorter ^1^H-^7^Li coordination distance than the other resolved acid species, but the relative concentration of these species vary with chain spacing. The r15AA copolymer and the r15AA-31%Li ionomer contain both an amorphous and crystalline phase, as reflected in the multiple *T*_g_/*T*_m_ transitions observed. These different phases are argued to explain the offset in the ^7^Li NMR chemical shifts between the precise and random materials. The *T*_g_ for the amorphous component in the r15AA-31%Li ionomer is the same as observed for the amorphous precise p9AA and p15AA Li ionomers (Table 1). If the local Li coordination was the only factor impacting *T*_g_, then differences in network structure created by ionic crosslinks should vary with acid group spacing and result in *T*_g_ variations. The consistent *T*_g_ between the different Li-ionomers suggest that a combination of both the Li coordination environment and the formation of different carboxylic acid hydrogen bonded complexes govern the observed *T*_g_. 

## 3. Experimental Section 

### 3.1. Ionomer Material Preparation 

The synthesis and characterization of the linear poly(ethylene-*co*-acrylic acid) co-polymers have been previously described [6]. Polymers with precisely spaced carboxylic acid groups were prepared using the ADMET chemistry, and polymers with randomly spaced carboxylic acid groups were obtained with the ROMP synthetic method. The unneutralized polymer materials are designated as p9AA, p15AA and r15AA, and correspond to samples with the carboxylic acid groups either precisely (p) located every 9th and 15th carbon along the backbone, or to samples where the acid groups are randomly (r) located on the polymer backbone, but with the average number of 15 carbons between the acid groups. The Li^+^ neutralized ionomers were prepared by dissolving the acid copolymer in a 1:4 mixture of 1,4-dioxane and 1-butanol at 90 °C, adding the appropriate amount of lithium acetate salt, followed by filtration of the resultant precipitant. These Li-neutralized materials are designated as p9AA-43%Li, p15AA-45%Li and r15AA-31%Li. The extent of Li^+^ neutralization achieved was determined using inductively coupled plasma elemental analysis performed by Galbraith Laboratories (Knoxville, TN, USA). The generalized structure for the random and precise and Li-exchanged P(E-AA) copolymers is shown in Chart 1. 

### 3.2. Solid-State ^1^H NMR Spectroscopy 

**Figure 11:**
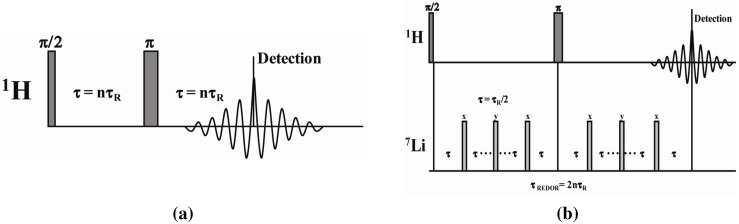
MAS NMR pulse sequences employed including the (**a**) rotor synchronized Hahn echo; and (**b**) the ^1^H-detected ^1^H-^7^Li REDOR sequence.

The solid-state 1D ^1^H magic angle spinning (MAS) NMR spectra were obtained on a Bruker AVANCE-III spectrometer operating at 600.13 MHz using a 2.5 mm broadband MAS probe, using N_2_ for spinning. A rotor-synchronized Hahn spin echo pulse sequence was employed (Figure 11a), with 2.5 µs π/2 pulse, 16 scan averages, and a 5 s recycle delay. The rotor spinning speed for analysis was 30 kHz, unless specifically noted. Spin regulation was maintained at ± 1Hz through the experiments. It is known that significant frictional heating occurs at high MAS speeds. The actual sample temperature was calibrated using the ^207^Pb chemical shift change of a secondary Pb(NO_3_)_2_ sample [28,29], with all temperatures reported in this paper reflecting this correction. The ^1^H MAS NMR chemical shifts were referenced to the secondary external standard adamantane, *δ* = +1.63 ppm with respect to TMS *δ* = 0.0 ppm.

**Figure 12:**
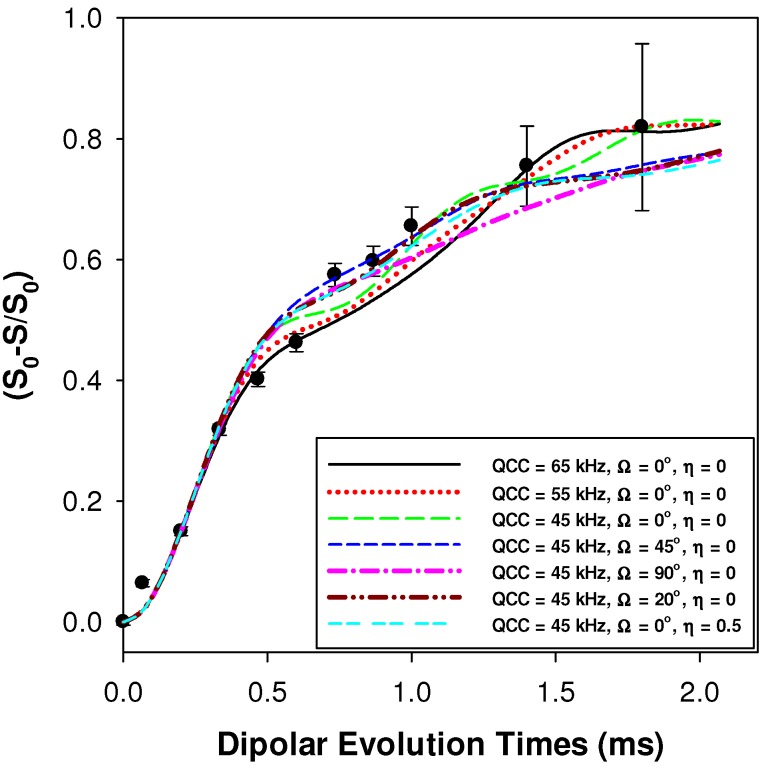
Predicted ^1^H-^7^Li REDOR buildup curves as a function of the quadrupolar coupling constant (QCC) and the relative orientation of the EFG and ^1^H-^7^Li dipolar tensors (Ω). The experimental REDOR response for the p9AA-43%Li ionomer is also shown (●). The ^1^H-^7^Li dipolar coupling constant was 1100 Hz for all simulations.

The ^1^H-^7^Li REDOR experiments were obtained using the sequence shown in Figure 11b [30]. The ^1^H-^7^Li REDOR buildup curve simulations were performed using the SIMPSON software package [31]. A series of different ^1^H-^7^Li dipolar coupling were analyzed to produce the different buildup curves shown in Figure 4 and Figure 5. All the REDOR simulations were scaled for the natural abundance of ^7^Li (92.58%). Multiple ^1^H-^7^Li couplings were not included in the simulations. For these simulation a ^7^Li quadrupolar coupling constant (QCC) of 65 kHz, an asymmetry parameter (η) of zero, and collinear dipolar and the quadrupolar electrical field gradient (EFG) tensors were assumed. The magnitude of the QCC was estimated from the spinning sideband manifold observed in the ^7^Li MAS NMR spectra. To address the impact of variations in the size of the QCC, or the relative orientation of the EFG and dipolar tensor a series of simulations were performed. Representative examples of the REDOR response are shown in Figure 12. For all parameter variation, the initial short time regime of the REDOR buildups overlap, with this region being dominated by the ^1^H-^7^Li dipolar coupling strength. At longer REDOR recoupling times, the buildup curves deviate from each other and show an increased dependence on the QCC and the relative tensor orientations (Ω). The dipolar coupling and the corresponding ^1^H-^7^Li distances were measured using these initial REDOR buildups.

### 3.3. MD Simulations 

Fully atomistic molecular dynamics simulations were carried out using the LAMMPS software [32], and the OPLS-aa force field [33], using a constant density and temperature (150 °C, 423 K), well above the glass transition temperature of these materials. The number of polymer chains in the simulations varied between 80 and 200 depending on the spacer length, with each chain containing four monomers. Polymers with a precise spacing of 9, 15 or 21 carbons were simulated at a Li-neutralization level of 43%. In the case of the p9AA polymer, additional neutralization levels were tested (10%, 25%, 75% and 100%). The molecular dynamics simulations are described in greater detail elsewhere [27]. 

### 3.4. Ab Initio Chemical Shift Calculations 

The small Li^+^(CH_3_COOH)_4_ clusters were optimized in the gas phase using the Gaussian 09 software [34] (Gaussian Inc., Wallingford CT) using the 6-311++G(2d,2p) basis set [35,36], and density functional theory (DFT) utilizing Becke’s three parameter exchange functional [37], and the LYP correlation function (B3LYP) [38]. To evaluate the variation in the chemical shield as a function of Li-O distance, structures were re-optimized assuming a fixed and equal bond distance (symmetric cluster). The chemical shielding tensors, σ, were calculated using the Gaussian 09 program utilizing the gauge-including atomic orbital (GIAO) method at the DFT level [39]. All NMR shielding calculations for the small Li^+^(H_2_O)*_n_* clusters used the same exchange and correlation functionals and basis sets as for the structure optimization. The NMR chemical shift of a species *i* is defined with respect to the chemical shielding of a reference species by

(5) $$\delta^{i}=\sigma_{\text{iso}}^{\text{ref}}-\sigma_{\text{iso}}^{i}$$

where positive *δ* values represent environments that are deshielded and resonate at a higher frequency. The shielding value of σ = +90.9 ppm, recently obtained from *ab initio* and MD simulations [26,40], was used for ^7^Li chemical shift referencing. A shielding of σ = +31.88 ppm for TMS was used to reference the ^1^H chemical shifts.

## 4. Conclusions 

Solid-state MAS NMR has been used to probe the lithium and carboxylic acid proton environments in partially neutralized polyethylene acrylic acid ionomers. These results reveal that distinct heterogeneous carboxylic acid structural motifs exist within the materials, while only a single Li environment was observed. These environments do not change significantly with spacing between acid groups or the degree of Li neutralization. Using REDOR NMR it was shown that the two different carboxylic acid proton environments result from different ^1^H-^7^Li coordination distances, and reflects the different acid coordination environments. MD simulations allowed the development of a structural motif that can explain these observations. The structure involves acid coordinated Li atoms that are in extended clusters. The majority of the unneutralized carboxylic acids are directly involved in the local Li coordination sphere, with both oxygen atoms coordinating to the Li, and the acid proton forming an additional hydrogen bond to oxygen. There is a minor population of protonated acid species that only contribute one oxygen to the Li coordination sphere. The NMR results also show that isolated, unneutralized, acid-acid dimer formation is not significant in these materials. 
